# Epigenetic Regulation of *BST-2* Expression Levels and the Effect on HIV-1 Pathogenesis

**DOI:** 10.3389/fimmu.2021.669241

**Published:** 2021-05-05

**Authors:** Ravesh Singh, Veron Ramsuran, Vivek Naranbhai, Nonhlanhla Yende-Zuma, Nigel Garrett, Koleka Mlisana, Krista L. Dong, Bruce D. Walker, Salim S. Abdool Karim, Mary Carrington, Thumbi Ndung’u

**Affiliations:** ^1^ HIV Pathogenesis Programme, Doris Duke Medical Research Institute, Nelson R Mandela School of Medicine, University of KwaZulu-Natal, Durban, South Africa; ^2^ Africa Health Research Institute (AHRI), Durban, South Africa; ^3^ Department of Microbiology, National Health Laboratory Services, KZN Academic Complex, Inkosi Albert Luthuli Central Hospital, Durban, South Africa; ^4^ School of Laboratory Medicine and Medical Sciences, College of Health Sciences, University of KwaZulu-Natal, Durban, South Africa; ^5^ The Ragon Institute of MGH, MIT and Harvard University, Cambridge, MA, United States; ^6^ Basic Science Program, Frederick National Laboratory for Cancer Research in the Laboratory of Integrative Cancer Immunology, Bethesda, MD, United States; ^7^ Centre for the AIDS Programme of Research in South Africa (CAPRISA), University of KwaZulu-Natal, Durban, South Africa; ^8^ Max Planck Institute for Infection Biology, Chariteplatz, Berlin, Germany; ^9^ Division of Infection and Immunity, University College London, London, United Kingdom

**Keywords:** *BST-2*, HIV-1, DNA methylation, epigenetic regulation, expression

## Abstract

HIV-1 must overcome host antiviral restriction factors for efficient replication. We hypothesized that elevated levels of bone marrow stromal cell antigen 2 (*BST-2*), a potent host restriction factor that interferes with HIV-1 particle release in some human cells and is antagonized by the viral protein Vpu, may associate with viral control. Using cryopreserved samples, from HIV-1 seronegative and seropositive Black women, we measured *in vitro* expression levels of *BST-2* mRNA using a real-time PCR assay and protein levels were validated by Western blotting. The expression level of *BST-2* showed an association with viral control within two independent cohorts of Black HIV infected females (r=-0.53, p=0.015, [n =21]; and r=-0.62, p=0.0006, [n=28]). DNA methylation was identified as a mechanism regulating *BST-2* levels, where increased *BST-2* methylation results in lower expression levels and associates with worse HIV disease outcome. We further demonstrate the ability to regulate *BST-2* levels using a DNA hypomethylation drug. Our results suggest *BST-2* as a factor for potential therapeutic intervention against HIV and other diseases known to involve *BST-2*.

## Introduction

To establish infection and replicate efficiently, HIV-1 must overcome host antiviral restriction factors. Host restriction factors that inhibit HIV-1 replication are an important component of the innate immune system that forms the first line of defense before adaptive immune responses are mobilized and established (1–4). *BST-2* (also termed *Tetherin/CD317/HM1.24*) was discovered as an anti-HIV host factor responsible for the prevention of virus release (5). Subsequently, additional mechanisms of HIV inhibition by *BST-2* have been reported (6) and studies have associated BST-2 expression levels with HIV viral control (6–8). Downregulation of BST-2 expression correlated with Vpu expression and elevated BST-2 induced a requirement for Vpu to facilitate HIV particle release in some cells (5, 9). Vpu promotes intracellular down-regulation of BST-2 (10, 11). However, BST-2 is an interferon-induced protein, which gets activated upon HIV infection (7, 12). Factors regulating the expression levels of the *BST-2* gene have not been fully resolved.

The “tethering” effect mediated by *BST-2* on HIV has subsequently been shown to restrict the replication of a diverse array of other enveloped viruses including other retroviruses, rhabdoviruses, alphaviruses, arenaviruses, filoviruses, herpesviruses, paramyxoviruses, orthomyxoviruses, orthohepadnaviruses and flaviviruses (12–21). In addition, BST-2 expression levels are elevated in several cancers such as head and neck, breast, cervical, lung, endometrial, myelomas, and glioblastomas (22–29) as well as lupus erythematosus (SLE) an autoimmune disease (30), suggesting that BST-2 could be an immunotherapeutic target for several diseases. If *BST-2* is directly affecting these conditions, then identifying the factors regulating *BST-2* expression could develop strategies against an array of diseases. DNA methylation has been linked with the regulation of *BST-2* expression particularly in cancer cells (24), and in lupus (30). A few human genes implicated in HIV control are known to be regulated by DNA methylation. Hypermethylation of FOXP3, EPB41L3, IL-2, CCR5 and HLA-A at gene regulatory sites, are associated with reduced gene expression and worse HIV outcome, whereas reduced methylation corresponds with increased expression of these genes and improved disease outcome (31–36). This highlights the potential importance of this epigenetic mode of gene regulation in HIV disease pathogenesis.

In this study, we found that increased BST-2 levels associated with HIV control. We further show DNA methylation as one of the regulatory mechanisms responsible for *BST-2* expression variation within HIV infected individuals. Furthermore, *BST-2* methylation levels correlate with HIV outcomes in both *ex vivo* and *in vitro* experiments, and experimental manipulation of *BST-2* methylation altered its expression levels. Together, these data suggest that manipulation of *BST-2* expression levels could be used as a therapeutic target for viral control.

## Materials and Methods

### Study Design

A chronic HIV infection cohort, Sinikithemba (SK; n=21) (37), was compared to the HIV negative arm of the acute infection cohort from the Females Rising through Education, Support, and Health (FRESH; n=65) study (38, 39) in a cross sectional analysis. We further studied a longitudinal HIV acute infection cohort, CAPRISA 002 (n=55) (40, 41), from pre-infection to >36 months of follow-up post HIV infection. All the samples used in this study were from South African females of Black ancestry. The study was approved by the Biomedical Research Ethics Committee of the University of KwaZulu-Natal.

### Sample Processing, Viral Load Quantification and CD4 Cell Enumeration

Peripheral blood mononuclear cells (PBMCs) were isolated within 6 hours of blood collection, and frozen in liquid nitrogen until use. Viral load was determined using the automated COBAS AMPLICOR HIV-1 Monitor Test v1.5 (Roche Diagnostics, Mannheim, Germany). CD4^+^ T cells were enumerated using the Multitest kit (CD4/CD3/CD8/CD45) on a four-parameter FACSCalibur flow cytometer (Becton Dickinson, San Jose, CA, USA).

### Real Time PCR Quantitation

RNA was extracted from 2 x 10^6^ PBMCs using the TRIzol LS Reagent (Invitrogen, Carlsbad, CA, USA). RNA from each sample was reversed transcribed using the iScript cDNA synthesis kit (Bio-Rad, California, United States of America). PCR primer and cycling conditions for *BST-2* and *GAPDH* (housekeeping gene) are available on request. *GAPDH* was used as reference gene (42). PCR-product amplification specificity was confirmed *via* melting curve analysis and agarose gel electrophoresis.

### Western Blotting

Cell lysates were boiled for 10 minutes in 4X Laemmli sample buffer (Bio-Rad), then separated by SDS-PAGE on 4 to 15% gels (Bio-Rad, California, United States of America) and transferred onto nitrocellulose membrane following standard methods. The membrane was then incubated with the primary antibody (rabbit monoclonal anti-BST-2 [cat. no. ab243229, Abcam, Cambridge, United Kingdom], and mouse polyclonal anti-alpha-tubulin [cat. no. ab7291, Abcam, Cambridge, United Kingdom]), diluted in 5% bovine serum albumin (BSA) (Roche, Basel, Switzerland) in tris-buffered saline and Tween 20 at a 1:100 or 1:5000 dilution overnight, followed by three washes in tris-buffered saline (TBS) and Tween 20 (TBST) for 10 minutes. The membrane was incubated with the secondary antibody (anti-rabbit, or anti-mouse) at a 1:20,000 dilution in 5% BSA in TBST for 1 hour on a rocker, followed by three washes in TBST for 10 minutes. Antibody-antigen complexes were detected *via* enhanced chemiluminescence reagents (SuperSignal West Dura extended-duration substrate, Thermo Scientific, Pierce Protein Research, United States of America). Proteins were visualized using the ChemiDoc XRS+ system with Image Lab software (Bio-Rad, California, United States of America).

### *In Vitro* HIV Infection

HIV-1 replication *in vitro* was assessed on PBMCs from 22 donors selected from the 65 healthy HIV uninfected individuals from the FRESH cohort. Individuals with the highest (n =11) and lowest (n=11) *BST-2* mRNA levels were included. PBMCs (2×10^6^/mL) were stimulated for 48 hours in R10 buffer [Roswell Park Memorial Institute (RPMI)-1640 medium (Grand Island, NY, USA) supplemented with 10% fetal calf serum (Hyclone Inc., Logan, UT, USA), gentamicin (Gibco-Brl, Gaithersburg, MD, USA) (100 mg/mL)] containing 5 mg/mL phytohemagglutinin (PHA) (Roche, Basel, Switzerland) and 5 mg/mL interleukin-2 (IL-2) (Roche, Basel, Switzerland). Following stimulation with PHA/IL-2, cells were washed with R10 buffer and then infected with HIV IIIB (NIH AIDS Reagent Repository) at 0.1 multiplicity of infection (MOI) by spinoculation (2 hours, 300 x *g* at 37°C). Infection was performed in a 24-well plate. Virus was subsequently removed by washing the cells, followed by cell culture for 7 days. *BST-2* mRNA expression levels and DNA methylation were analyzed by real-time PCR and pyrosequencing. Supernatants from days 2, 4 and 7 were harvested and analyzed by p24 antigen capture enzyme-linked immunosorbent assay (ELISA [Biomérieux, Marcy-l’Étoile, France]).

### DNA Methylation by Sequencing

Primer design for the detection of methylation within the *BST-2* promoter region was performed using MethPrimer online software, default settings (43) (Forward meth primer 1 GGTTAGTTTTTGTTGTAGGAGATGG; Reverse meth primer 1 AACTATTACAAAATACCCATAAAAAAC; Forward meth primer 2 TTGATGGTGAAGATAATTAAGGGTATT; Reverse meth primer 2 AAAAACTACTAATCAAAACACTTCCTAAAA). Sodium bisulphite conversion was performed on genomic DNA extracted from PBMCs using the EZ DNA methylation™ kit (Zymo Research, Irvine, USA). Using the *BST-2* specific primers on the bisulphite converted DNA, a PCR was run using the following conditions (95°C for 15 minutes, 45 cycles of 95°C for 30 seconds, 60°C for 45 seconds, 72°C for 30 seconds and one cycle of 72°C for 10 minutes). The level of methylation at specific sites within the *BST-2* promoter was measured using pyrosequencing (Roche, Basel, Switzerland).

### 5’-aza-2-deoxycytidine Treatment

Treatment of cells with the DNA hypomethylation drug, 5’-aza-2-deoxycytidine (5’-Aza-CdR), was performed as previously described (32). Briefly, PBMCs from healthy donors (n=40) were treated with 10 µM 5’-Aza-CdR (Sigma, St. Louis, United States America) or with dimethyl sulfoxide (DMSO; treatment control) for 24 hours at 37°C. *BST-2* mRNA levels from 5’-Aza-CdR treated cells were compared to DMSO treated and then plotted against the untreated mRNA levels.

### Statistical Analysis

Statistical analyses were conducted using Instat Graphpad Prism V.5 and SAS version 9.4. All expression data was log_10_ transformed to ensure normality (44, 45). Gene expression levels between HIV negative and HV infected donors were compared using an unpaired t-test. *BST-2* mRNA expression levels for HIV positive donors prior to infection and at three months post-infection were compared using paired t-test. Furthermore, we calculated the Pearson correlation coefficient to measure the strength of an association between *BST-2* mRNA expression levels and methylation at each time-point.

Univariable linear mixed model with autoregressive order one covariance structure were fitted to determine if there was an association between *BST-2* gene expression and viral load. In this model, we included a random effect for the participant or subject.

## Results

### *BST-2* mRNA Expression Level Associates With HIV Viral Control

**Figure 1:**
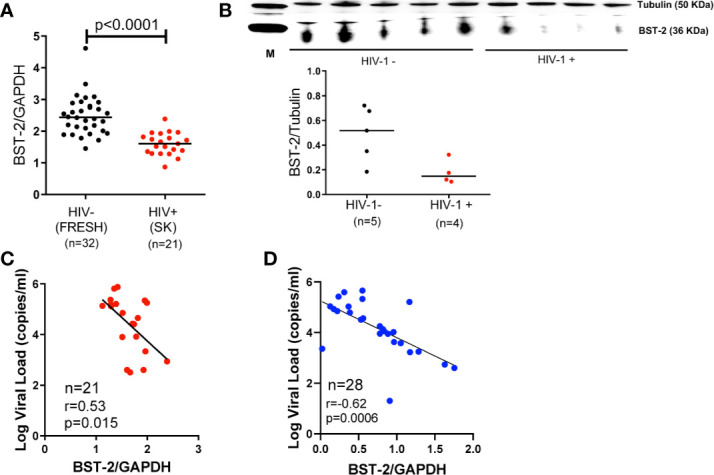
*BST-2* mRNA and protein expression levels within HIV-negative and positive individuals. **(A)** Comparison of *BST-2* mRNA expression levels measured in HIV negative and positive donors from the FRESH, (black dots), and SK, (red dots) cohorts, respectively. Significantly elevated *BST-2* levels are found within HIV negative donors *vs.* positives (p<0.0001). These represent unmatched donors from two separate cohorts. The HIV positive donors are ARV naïve chronically infected. **(B)** Protein levels of BST-2 were measured on 5 HIV negative donors and 4 HIV infected donors from the FRESH and SK cohorts, respectively. BST-2 protein levels were assessed using a Western blot assay. The levels of HIV infected donors are lower than the HIV negative. **(C)** *BST-2* mRNA expression levels were correlated with log viral load within the SK cohort. Higher mRNA levels correlated with lower log viral load levels (r=-0.53, p=0.0150). **(D)** A negative correlation was also observed when examining the effect of *BST-2* mRNA expression levels and viral load using the CAPRISA 002 cohort at the >36 month time point (r=-0.62, p=0.0006).

We investigated the effect of HIV infection on *BST-2* mRNA expression levels in PBMCs *ex vivo*. We found significantly higher levels of *BST-2* in HIV negative individuals (n=32, FRESH cohort, black dots) compared to HIV infected late stage antiretroviral (ARV)-naïve individuals (n=21, SK cohort, red dots; p < 0.0001; **Figure 1A**). To validate these findings for consistency of mRNA expression with protein levels, we randomly selected donors, based on sample availability, from 5 HIV negative donors and 4 HIV infected donors, which formed subsets of the FRESH and SK cohorts, respectively. Western blot assays showed consistent BST-2 protein expression levels relative to mRNA expression levels, with protein expression higher in HIV- compared to HIV+ donors (**Figure 1B**). We next explored the relationship between *BST-2* mRNA expression levels and HIV-1 viral load. A negative correlation was observed in both SK (r=-0.53, p=0.015; **Figure 1C**), and CAPRISA 002 cohorts (n=28, r=-0.62, p=0.0006; **Figure 1D**), all individuals analysed cross-sectionally were past 36 months post infection in both cohorts.

*BST-2* mRNA levels and viral load were also tested longitudinally at three timepoints (3, 12 and >36 months) in the CAPRISA 002 cohort. The results of the generalized estimating equation (GEE) model revealed consistent results to the cross-sectional data where higher mRNA levels associated with decreased viral load (Effect = -0.022; Standard error = 0.009; p=0.0003).

### Effect of *BST-2* DNA Methylation on *BST-2* Expression and HIV Disease

**Figure 2:**
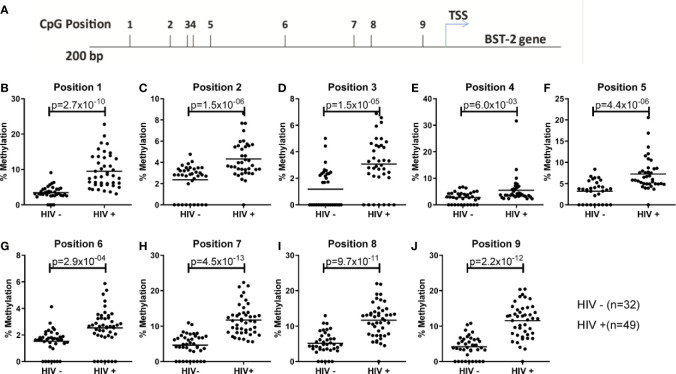
Examining DNA methylation levels across unmatched HIV uninfected and infected donors. **(A)** Location of nine CpG sites within the *BST-2* promoter region 200bp upstream of the TSS. **(B-J)** Using HIV negative (FRESH) and HIV positive (SK) cohorts the percentage methylation, using pyrosequencing of bisulfite converted DNA, was calculated for each of the nine sites.

The inverse effect of DNA methylation on *BST-2* expression has been shown previously in the context of cancer and autoimmune studies (24, 30). Here, we examined the effect DNA methylation on *BST-2* expression levels within an HIV setting. Nine CpG sites located within 200 bp of the transcription start site were evaluated for methylation levels (**Figure 2A**) in HIV positive and negative individuals (SK *vs.* FRESH cohort respectively). All sites showed significantly higher methylation levels within the HIV infected group (**Figures 2B–J**), suggesting that increased *BST-2* methylation levels in chronic HIV infection results in decreased expression level of the gene as observed in **Figure 1**.

**Figure 3:**
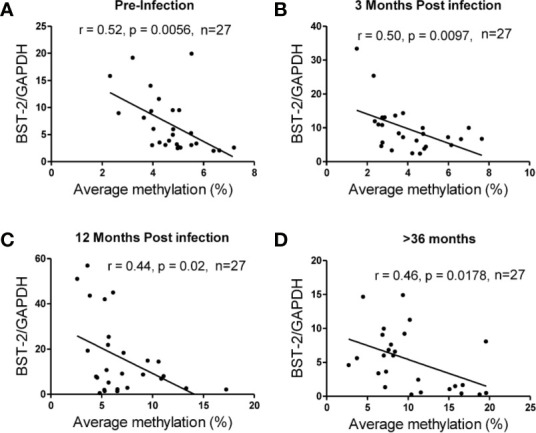
Correlation of DNA methylation and *BST-2* mRNA expression levels across HIV disease. Average methylation was calculated as the average methylation level across nine sites within 200bp upstream of the transcription start site. A strong negative correlation was observed at each of the time points examined for a set of n=27 matched samples at varying time points across disease progression. **(A)** pre-infection (r=-0.52, p=0.0056), **(B)** 3 months’ post infection (r=-0.50, p=0.0097), **(C)** 12 months’ post infection (r=-0.44, p=0.02) and **(D)** >36 months (r=-0.46, p=0.0178).

The average methylation across the nine CpG sites was compared to *BST-2* mRNA expression levels in samples with four different HIV serostatus or disease stages, i.e. pre-infection, 3, 12- and >36-months’ post-infection using n=27 matched ARV-naïve samples, based on sample availability. An inverse correlation was observed at all time points; pre-infection (r=-0.52, p=0.0056; **Figure 3A**), 3 months (r=-0.50, p=0.0097; **Figure 3B**), 12 months (r=-0.44, p=0.02; **Figure 3C**) and >36 months (r=-0.46, p=0.0178; **Figure 3D**). These data strongly point to methylation as a major contributor in regulation of *BST-2* expression levels.

**Figure 4:**
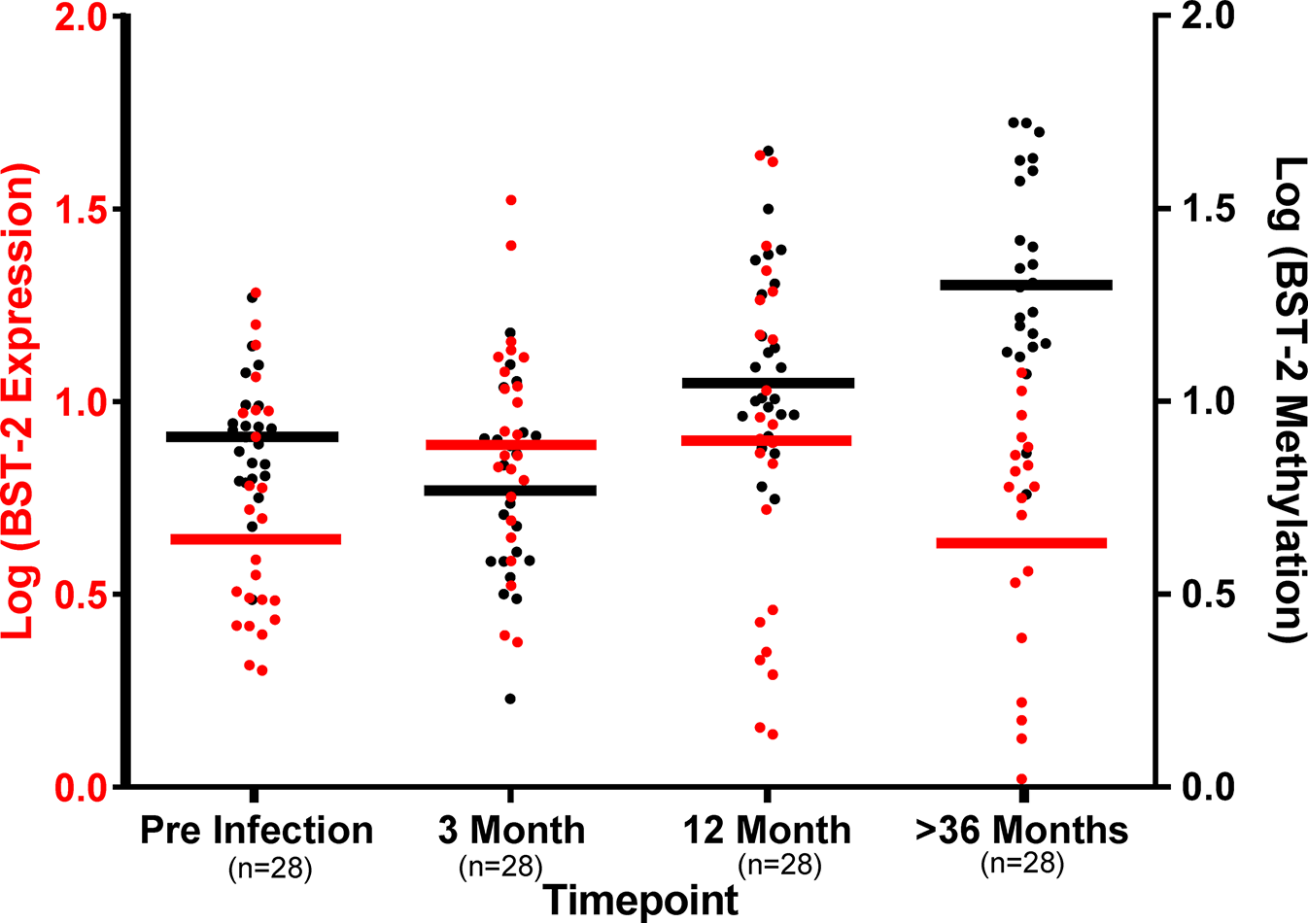
DNA methylation levels dictate *BST-2* mRNA levels during HIV disease. Baseline levels of 27 matched samples at varying time points across disease progression show at the pre-infection higher methylation (Black) and low *BST-2* expression (Red), while at acute infection (3-month post infection) methylation and expression levels are at similar level, due to IFN induction. The *BST-2* expression and methylation levels invert at 12 months’ post infection. The most dramatic difference is observed at the >36 months timepoint, these individuals are at a chronic phase of infection, at this time point we observe the lowest expression and highest methylation.

Comparison of *BST-2* DNA methylation pattern with mRNA expression levels indicate distinctions at the four timepoints. At the pre-infection time point, *BST-2* expression levels are relatively low, with modest methylation of the gene. Three months after HIV infection, *BST-2* expression levels increase with a concomitant decrease in methylation, perhaps as a result of the immune response in acute infection, including IFN-I production, which is known to enhance *BST-2* production (3) (**Figure 4**). Methylation begins to increase at 12 months’ post infection, and by 36 months post infection, the mean expression of *BST-2* dips to pre-infection levels while methylation is considerably higher than that at pre-infection timepoint (**Figure 4**). Overall, these results suggest that DNA methylation is not the sole contributor to BST-2 expression variation.

### *In Vitro* HIV Infection and 5’-Aza-CdR Treatment

**Figure 5:**
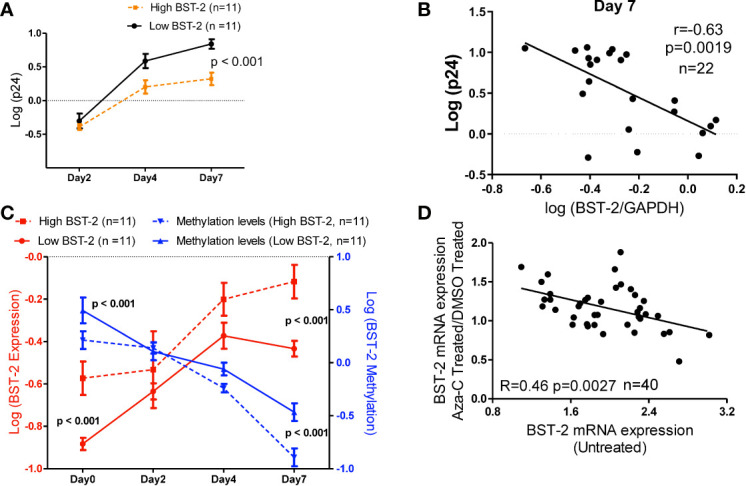
*BST-2* mRNA expression and methylation levels correlate in an *in vitro* viral replication assay, and treatment with a DNA hypo-methylation drug increases *BST-2* mRNA expression levels. **(A)** Individuals were pre-selected based on *BST-2* expression levels for a HIV replication assay. PBMCs from HIV negative donors (n=22) were infected with HIV IIIB viral strain, the amount of virus present was determined by measuring the p24 antigen using an ELISA assay. Measurements of p24 for both high and low *BST-2* donors were taken at days 2, 4 and 7. Donors with higher *BST-2* levels (dotted line) had lower level of p24, while donors with lower *BST-2* levels (solid line) had significantly higher p24 levels (p<0.001). **(B)** A negative correlation was observed when comparing the HIV replication levels against the BST-2 mRNA levels at day 7 from the *in vitro* HIV infection assay (r=-0.63, p=0.0019). **(C)** mRNA and DNA were used to measure *BST-2* expression (red) and methylation levels (blue), respectively, from high and low *BST-2* donors (n=22) at four time points during the viral replication assay, days 0, 2, 4 and 7. Within high *BST-2* donors, we find high expression (red dotted line) associated with lower methylation (blue dotted line) and vice versa for low *BST-2* levels, where low expression (red solid line) associated with higher methylation (blue solid line). **(D)** PBMCs from HIV negative donors (n=40) were split into three subsets; the first subset was treated with a DNA methyltransferase inhibitor that causes hypomethylation (5’-Aza-CdR), while the second subset was treated with DMSO. Both subsets were incubated for 24 hours. *BST-2* mRNA expression from 5’-Aza-CdR and DMSO treated cells were compared and plotted as a fold change against *BST-2* mRNA from an untreated time point (third subset), a significant correlation was observed (R=-0.46, p=0.0027).

We next examined the impact of *BST-2* mRNA expression levels on HIV replication *in vitro*. HIV replication was assessed by the amount of p24 released into tissue culture supernatant following infection of PBMCs from HIV negative individuals either having the highest (n=11) or lowest (n=11) *BST-2* mRNA expression levels screened from a cohort of 65 HIV negative donors. p24 measurements, taken at days 2, 4 and 7, showed that individuals with higher *BST-2* expression (dotted line, **Figure 5A**) significantly associated with lower viral replication, at days 4 and 7 post infection, compared to lower *BST-2* expression individuals (solid line, **Figure 5A**; ANOVA, p<0.001). Further, a negative correlation between HIV replication and *BST-2* mRNA expression levels was observed at day 7 (r=-0.63, p=0.0019, **Figure 5B**). These data support a model in which higher *BST-2* levels diminish HIV replication.

Next, we tested whether DNA methylation correlated with *BST-2* mRNA expression levels in an *in vitro* HIV infection assay. Individuals with high *BST-2* expression levels (red dotted line, **Figure 5C**) possessed low methylation levels (blue dotted line) measured at days 0, 2, 4 and 7 days post HIV infection. Conversely, low *BST-2* mRNA expression donors (red solid line, **Figure 5C**) associated with high methylation levels (blue solid line) throughout the time course. Further, the overall difference between the methylation levels within donors either possessing high or low *BST-2* expression levels was significant (**Figure 5C**; ANOVA, p<0.001). Thus, *BST-2* mRNA expression levels associate with the level of *BST-2* DNA methylation, even within an *in vitro* time course of HIV infection.

5’-Aza-CdR induces hypomethylation due to its ability to inhibit DNA methyltransferases, the enzymes responsible for methylation. As manipulation of *BST-2* expression could be considered as a therapeutic intervention in HIV disease, we tested whether 5’-Aza-CdR enhanced *BST-2* expression differentially among donors as a function of the intrinsic expression level of *BST-2*. *BST-2* levels were measured from HIV negative healthy donor PBMCs (n=40) treated with either 5’-Aza-CdR or DMSO (to measure baseline potential for stimulation in each subject). *BST-2* ratios of 5’-Aza-CdR/DMSO treated mRNA levels were then plotted against the *BST-2* levels measured in corresponding untreated PBMCs (**Figure 5D**). A negative correlation between levels of *BST-2* mRNA expression in untreated and Aza-CdR treated PBMC (R=-0.46, p=0.0027; **Figure 5D**) was observed. Donors with the lowest intrinsic (i.e. untreated) *BST-2* mRNA expression levels had the greatest increase in mRNA expression following 5’-Aza-CdR treatment. These data point directly to DNA methylation as a primary factor in regulating *BST-2* gene expression. Increasing *BST-2* gene expression by demethylation may therefore enhance resistance to HIV, given the observation that higher *BST-2* expression associates with HIV control.

## Discussion

Here we show that expression levels of *BST-2*, a potent antiviral cellular protein are negatively associated with viral loads in an antiretroviral-naive cohort of women followed longitudinally from acute HIV-1 infection. Similar results were obtained from an ART-naive chronically infected cohort of participants with unknown time of infection. *BST-2* levels are lower in chronically infected HIV individuals compared to uninfected persons, however in longitudinally followed matched samples, *BST-2* levels first increase significantly over baseline and then decline slowly. We showed *BST-2* expression and DNA methylation levels within the gene promoter region are negatively correlated. These findings are consistent in HIV infected subjects in studies performed *ex vivo* and *in vitro*. Moreover, we pharmacologically altered *BST-2* expression levels by manipulating methylation levels with 5’-Aza-CdR, leading to an increase in *BST-2* mRNA expression, especially within cells with lower intrinsic *BST-2* levels.

*BST-2* levels have been shown to inhibit the production of HIV-1 particles by hindering the release of virion progeny (5, 46). However, HIV-1 has developed the ability to counteract this mechanism through the accessory viral protein, Vpu. BST-2 is trafficked from the viral budding sites on the cell surface by a Vpu-mediated mechanism, which thereafter sequesters the host protein to a perinuclear compartment (47). Vpu-null or defective viruses are most prone to BST-2-mediated inhibition. Previous studies have demonstrated that BST-2 surface levels are elevated during acute infection and then progressively decrease throughout the stages of infection, even after initiation of ART (7, 8). In line with these findings, we observed an elevation in mRNA expression of *BST-2* during acute infection both *ex vivo* and *in vitro*, with a subsequent decrease observed by 36 months post HIV-infection. The plasticity of *BST-2* methylation observed suggests that methylation levels are a strong regulator of *BST-2* expression even within a disease setting, although the mechanism regulating the methylation levels requires investigation.

Due to sample availability, we used bulk PBMCs to measure *BST-2* mRNA expression, rather than CD4+ T cells specifically. A previous study measuring cell surface BST-2 showed no differences in expression patterns between individual cells types, PBMCs, mononuclear leukocytes, including CD4-positive, CD8-positive T lymphocytes, B cells, across stages of HIV infection (7). Although the level of mRNA expression does not always reflect protein expression levels, our Western blot assay in a small number of participants suggested a fair correlation. Sample limitations prevented us from examining the correlation between BST-2 mRNA levels and cellular surface expression, however, previous studies have demonstrated the correlation between these subsets (24, 48, 49). These studies have shown that BST-2 mRNA and protein levels correlate in mice, monkeys and humans. Furthermore, the studies also show specific tissues and cell types have strong correlations. The effect is observed across diseases (cancer, SIV, and Mouse mammary tumor virus) and healthy human controls (24, 48, 49).

It is plausible that other human HIV restriction factors could be regulated through DNA methylation. Each factor contributing toward the overall HIV disease outcome. Whole genome methylation analysis on a pair of monozygotic twins with discordant HIV status found several distinct differential methylation regions in the HIV infected twins (50, 51). Furthermore genome-wide methylation analysis of 85 unrelated individuals with varying HIV statuses showed differential genome-wide patterns which was associated with their ability to control HIV replication (52). Future studies should focus on larger cohorts of monozygotic twins or consider longitudinal studies such that the changes in DNA methylation profiles may be followed up at the different time points of HIV infection.

DNA methylation is just one of the mechanisms contributing to the variation in *BST-2* expression levels. Another mechanism identified is a proposed regulatory variant, rs12609479, located in the *BST-2* promoter region, which associated with decreased risk of acquiring HIV-1. The rs12609479-A allele associated with increased BST-2 expression and decreased risk of acquiring HIV-1 (53, 54). The 9 CpG sites that were examined in this study did not contain any polymorphisms and rs12609479 was not located in a CpG site. Despite rs12609479 not being affected by methylation, previous studies have shown diverse changes with respect to minor allele frequency across various ethnic groups (55, 56). Future studies are required to fully understand all the contributing factors responsible for BST-2 expression variation including methylation status across various ethnic groups. Despite these limitations, we found a reproducible association of BST-2 mRNA expression levels with HIV control. Our results were further validated with *in vitro* data.

In conclusion, we reproducibly demonstrate *BST-2* expression levels associate with HIV viral control within a high disease burden setting. DNA methylation was shown to regulate *BST-2* levels and observed to associate with HIV disease. The use of the demethylating drug 5’-Aza-CdR *in vitro* resulted in increased *BST-2* expression levels among donors with low baseline expression levels. Thus, HIV control through higher *BST-2* expression levels, as determined in part by decreased methylation, may suggest strategic mechanisms for HIV cure therapy.

## Data Availability Statement

The raw data supporting the conclusions of this article will be made available by the authors, without undue reservation.

## Ethics Statement

The studies involving human participants were reviewed and approved by Biomedical Research Ethics Committee of the University of KwaZulu-Natal. The patients/participants provided their written informed consent to participate in this study.

## Author Contributions

RS, VR, MC and TN conceptualized the study. RS, VR, TN, NG, KM, KD, BW, and SK assisted with the cohort setup and proposal design. RS and VR performed the laboratory work. RS, VR, VN, and NY-Z performed the data analysis. RS, VR, MC and TN wrote the paper. All authors contributed to the article and approved the submitted version.

## Funding

This study was supported by the South African Department of Science and Innovation/National Research Foundation Research Chairs Initiative (SARChI) grant to TN, a grant from the Swiss South Africa Joint Research Programme (SSAJRP) to TN. Partial funding for this work was received from the Gates Foundation, the International AIDS Vaccine Initiative (IAVI) (UKZNRSA1001) and Gilead Sciences (grant ID #00406). RS was supported by the Columbia University-Southern African Fogarty AIDS International Discovery and Research Program (AITRP) through the Fogarty International Center, National Institutes of Health (grant # D43TW000231). VR is a FLAIR Research Fellow (FLAIR Fellowship programme is partnership between the African Academy of Sciences and the Royal Society that is funded by the UK Government as part of the Global Challenge Research Fund [GCRF]), and was supported by the South African Medical Research Council (SAMRC) with funds from the Department of Science and Technology. This work was also supported in part through the Sub-Saharan African Network for TB/HIV Research Excellence (SANTHE), a DELTAS Africa Initiative [grant # DEL-15-006]. The DELTAS Africa Initiative is an independent funding scheme of the African Academy of Sciences (AAS)’s Alliance for Accelerating Excellence in Science in Africa (AESA) and supported by the New Partnership for Africa’s Development Planning and Coordinating Agency (NEPAD Agency) with funding from the Wellcome Trust [grant # 107752/Z/15/Z], the Africa Health Research Institute (AHRI) Wellcome Strategic Core award [grant # 201433/Z16/Z] and the UK government. The views expressed in this publication are those of the author(s) and not necessarily those of AAS, NEPAD Agency, Wellcome Trust or the UK government. This project has been funded in whole or in part with federal funds from the Frederick National Laboratory for Cancer Research, under Contract No. HHSN261200800001E. The content of this publication does not necessarily reflect the views or policies of the Department of Health and Human Services, nor does mention of trade names, commercial products, or organizations imply endorsement by the U.S. Government. This Research was supported in part by the Intramural Research Program of the NIH, Frederick National Lab, Center for Cancer Research.

## Conflict of Interest

The authors declare that the research was conducted in the absence of any commercial or financial relationships that could be construed as a potential conflict of interest.

The handling Editor declared a shared affiliation, though no other collaboration, with one of the authors [MC].
